# Supplementary material for: Neonate Bloodstream Infections in Organization for Economic Cooperation and Development Countries: An Update on Epidemiology and Prevention
Source: J Clin Med. 2019 Oct 21;8(10):1750. doi: 10.3390/jcm8101750 (PMC6832148; doi:10.3390/jcm8101750)
Supplement: Supplementary file 1 [file jcm-08-01750-s001.pdf]

Table S1, Supplementary material: The study selection process and search strategy.

Country	Preselection, No of records		Proper selection: review of titles and abstracts	Qualified for this review
	searching database with keywords: „neonatal bloodstream infection”+ country	searching database with keywords „neonatal sepsis” + country		
Australia	39	277	12	3
Austria	8	48	3	1
Belgium	20	57	3	1
Canada	61	241	11	1
Chile	7	25	1	1
The Czech Republic	0	19	0	0
Denmark	16	36	4	2
Estonia	5	15	3	1
Finland	17	34	2	1
France	46	166	9	2
Germany	51	207	11	2
Greece	15	61	4	1
Hungary	9	10	1	0
Iceland	2	1	0	0
Ireland	8	48	1	1
Israel	25	143	10	3
Italy	55	266	9	1
Japan	29	76	4	1
Korea	12	60	3	1
Latvia	1	1	0	0
Lithuania	2	4	0	0

Luxembourg	0	1	0	0
Mexico	12	42	2	1
The Netherlands	36	209	10	1
New Zealand	6	49	4	2
Norway	9	40	3	2
Poland	11	31	3	2
Portugal	10	24	0	0
The Slovak Republic	1	0	0	0
Slovenia	1	6	0	0
Spain	32	146	8	1
Sweden	14	82	4	1
Switzerland	18	97	3	1
Turkey	29	224	9	1
The United Kingdom	68	250	14	1
The United States of America	247	953	48	2
